# Bioremediation of Agricultural Soils Polluted with Pesticides: A Review

**DOI:** 10.3390/bioengineering8070092

**Published:** 2021-07-02

**Authors:** Carla Maria Raffa, Fulvia Chiampo

**Affiliations:** Department of Applied Science and Technology, Politecnico di Torino, Corso Duca degli Abruzzi 24, 10129 Torino, Italy; carla.raffa@polito.it

**Keywords:** pesticides, bioremediation, agricultural soil, environmental pollution, sustainable agriculture, toxicity, health effects

## Abstract

Pesticides are chemical compounds used to eliminate pests; among them, herbicides are compounds particularly toxic to weeds, and this property is exploited to protect the crops from unwanted plants. Pesticides are used to protect and maximize the yield and quality of crops. The excessive use of these chemicals and their persistence in the environment have generated serious problems, namely pollution of soil, water, and, to a lower extent, air, causing harmful effects to the ecosystem and along the food chain. About soil pollution, the residual concentration of pesticides is often over the limits allowed by the regulations. Where this occurs, the challenge is to reduce the amount of these chemicals and obtain agricultural soils suitable for growing ecofriendly crops. The microbial metabolism of indigenous microorganisms can be exploited for degradation since bioremediation is an ecofriendly, cost-effective, rather efficient method compared to the physical and chemical ones. Several biodegradation techniques are available, based on bacterial, fungal, or enzymatic degradation. The removal efficiencies of these processes depend on the type of pollutant and the chemical and physical conditions of the soil. The regulation on the use of pesticides is strictly connected to their environmental impacts. Nowadays, every country can adopt regulations to restrict the consumption of pesticides, prohibit the most harmful ones, and define the admissible concentrations in the soil. However, this variability implies that each country has a different perception of the toxicology of these compounds, inducing different market values of the grown crops. This review aims to give a picture of the bioremediation of soils polluted with commercial pesticides, considering the features that characterize the main and most used ones, namely their classification and their toxicity, together with some elements of legislation into force around the world.

## 1. Introduction

Soil pollution is a worldwide problem that draws its origins from anthropologic and natural sources. Urbanization, industrialization, and food-demand increases have required the use of compounds, substances, and chemical agents, which, over the years, have brought on the dispersion and accumulation of pollutants in the environment. The common pollutants present in the soil are heavy metals, polycyclic aromatic hydrocarbons (PAHs), or pesticides [1].

Pesticides are chemical compounds used to eliminate pests. They are chemical or biological agents, that weaken, incapacitate, and kill pests. Based on the types of targeted pests, the pesticides can be divided into several groups, namely insecticides, herbicides, rodenticides, bactericides, fungicides, and larvicides.

During the 19th and 20th centuries, the extracts from plants, namely pyrethrins, were used as insecticides, fungicides, and herbicides. The increase in pesticide use happened with synthetic chemistry during the 1930s. In this period, inorganic chemicals such as arsenic and sulfur compounds were applied for crop protection. The arsenic poison was fatal to insects, while the sulfur was used as a fungicide. At the beginning of the Second World War, numerous pesticides were synthesized, mainly organic chemicals, such as dichlorodiphenyltrichloroethane (DDT), aldrin, and dieldrin used as insecticides, while 2-methyl-4-chlorophenoxyacetic acid (MCPA) and 2,4-dichlorophenoxyacetic acid (2,4-D) were used as herbicides [2].

After 1945, there was a rapid development of the agrochemical field, characterized by the introduction of many insecticides, fungicides, herbicides, and other chemicals, to control pests and ensure the yields of agricultural production. Moreover, pesticides are applied in aquaculture, horticulture, and for various general household applications. They are also used to control vector-borne diseases (e.g., malaria and dengue) [3].

**Figure 1:**
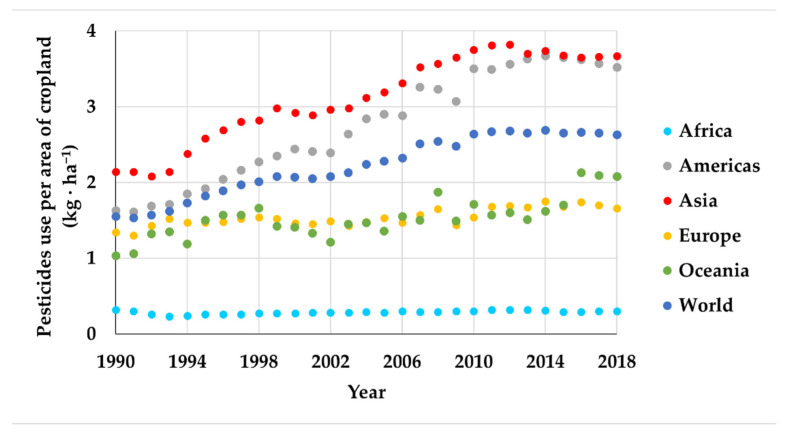
Pesticides use per area of cropland (data from [4]).

**Figure 2:**
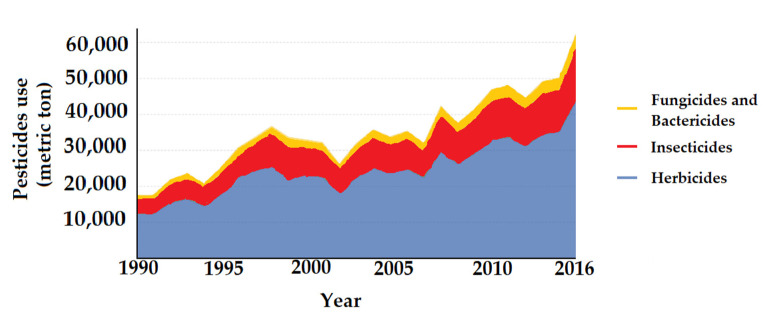
Pesticides use from 1990 to 2016 (data from [4]).

From 1990 to 2018, there have been registered amounts of used pesticides by all countries in the world, especially in Asia and America. The world average quantity has increased from 1.55 kg·ha^−1^ in 1990 to 2.63 kg·ha^−1^ in 2018, as shown in Figure 1. Looking at the types, fungicides and bactericides are used more than the others (Figure 2).

There has been no decrease even over the years, and directives have been implemented in many countries around the world to reduce the use of pesticides, for example, the Regulation (EC) 1107/2009 [5] of the European Union or the Stockholm Convention [6], which focuses on eliminating or reducing of persistent organic pollutants (POPs). To this purpose, the governments have to take measures to eliminate or reduce the release of POPs into the environment.

When pesticides are used, a part of them remains in the soil, and the accumulation affects the microorganisms living there. Human exposure can occur through the ingestion of pesticide-contaminated water and food, the inhalation of pesticide-contaminated air, and directly from occupational, agricultural, and household use. The pesticides can enter the human body by dermal, oral, eye, and respiratory pathways [7]. The toxicity of pesticides depends on the electronic properties and the structure of the molecule, dosage, and exposure times [8,9].

For these reasons, the residual pesticide concentration present in the soil must be reduced, and effective remediation techniques must be used to do this. An ecofriendly, cost-effective, rather efficient method is bioremediation, which is an alternative to more expensive and toxic approaches, such as chemical and physical methods. In biodegradation, the removal can be achieved by exploiting the microbial activity of microorganisms. The microorganisms, primarily bacteria [10], or fungi [11] transform pesticides into less complex compounds, CO_2_, water, oxides, or mineral salts, which can be used as carbon, mineral, and energy source. In these reactions, the enzymes have an important role since they act as catalysts [12].

Several techniques are available for the biodegradation of pesticides, which could develop in aerobic or anaerobic conditions based on types of microorganisms. Moreover, the bioremediation techniques can be divided into three categories depending on where the remediation treatment is done, namely in situ, ex situ, or on-site.

In the in situ approach, the treatment is involved in the contaminated zone, and usually, the process is aerobic. The main in situ techniques are natural attenuation, bioaugmentation, biostimulation, bioventing, and biosparging. In the ex situ methods, the contaminated soil is removed from polluted sites and transported to other places for treatment. Bioreactors, composting, landfarming, and biopiles are ex situ treatments. The on-site approach consists of the treatment of polluted soil on the surrounding site, to say the soil is removed from its original position but cleaned up in the neighborhood without any impact due to its transport.

In the literature, several reviews on pesticides have been published in the past years. Each of them is mainly focused on one topic. However, by this approach, the knowledge of the pesticide sector and its problems is lacking. Table 1 reports a shortlist of these publications.

The current review aims to give an overview of the bioremediation of soils polluted with commercial pesticides, considering the features that characterize the main and most used ones, namely, their classification and their toxicological issues, together with some elements of legislation into force around the world.

## 2. Classification of Pesticides

The pesticides can be classified by different criteria such as chemical classes, functional groups, mode of action, and toxicity.

### 2.1. Classification by Origin

The pesticides can be classified by their origin, namely chemical pesticides and biopesticides. Chemical pesticides are generally organic compounds that can have natural sources or by chemical synthesis [7]. Biopesticides are natural substances that control pests by nontoxic mechanisms [25].

#### 2.1.1. Classification by Chemical Composition

**Figure 3:**
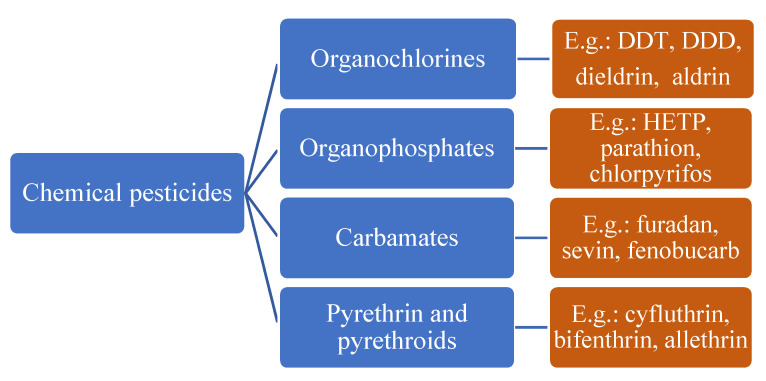
Classification of the chemical pesticides. DDT: dichlorodiphenyltrichloroethane; DDD: dichlorodiphenyldichloroethane; HETP: hexaethyl tetraphosphate.

With this classification, four main groups can be identified: organochlorines, organophosphates, carbamates, and pyrethrins and pyrethroids (Figure 3 and Table 2). The information on the chemical and physical characteristics of pesticides is very useful in determining the mode of application, precautions that need to be taken during the application, and the application rates [26].

##### Organochlorines

Organochlorine pesticides (OCs) are organic compounds, namely hydrocarbon chains bonded with at least one covalently bonded atom of chlorine (Table 2).

These compounds are widely used in agriculture, especially as insecticides to control a broad range of insects. The most common organochlorines are dichlorodiphenyltrichloroethane (DDT), dichlorodiphenyldichloroethane (DDD), dicofol, dieldrin, lindane, aldrin, chlordane, endosulfan, isodrin, isobenzan, toxaphene, and chloropropylate [27].

These compounds are lipophilic and are difficult to decompose, thus tending to bioaccumulate in tissues and remaining in the environment. For their high persistence in the environment, OCs belong to the class of persistent organic pollutants (POPs). They may cause damage to living beings, causing mutagenic effects, histopathological effects, enzyme-inducing and/or enzyme-inhibiting, carcinogenicity, and teratogenicity [28].

For human health, organochlorine exposure may increase the risk of breast cancer [29,30].

##### Organophosphates

Organophosphates (OPs) are synthetic pesticides, which include phosphoric acid esters or thiophosphoric acid esters. The general structure is reported in Table 2.

Hexaethyl tetraphosphate (HETP) was the first one synthesized and used as agricultural insecticides. OPs are acutely toxic for insects and other animals, including birds, amphibians, and mammals. The cause of their toxicity is due to inhibition of the acetylcholinesterase (AChE) in the central and peripheral nervous system [31,32].

The inhibition of this enzyme causes muscarinic and nicotinic effects. Muscarinic symptoms are linked to the assumption system:

- for inhalation, the symptoms may be cough, expectoration of frothy secretions, chest tightness, and wheeze, pulmonary edema;
- for ingestion hypersalivation, nausea, vomiting, abdominal cramps, diarrhea, and tenesmus;
- for eye, miosis, blurred vision, and eye pain.

Nicotinic effects are profuse sweating, fasciculation, progressive flaccidity, and weakness of proximal muscle groups, such as the neck flexors, then the extraocular muscles and muscles of respiration [33].

##### Carbamates

Carbamates are compounds derived from carbamic acid. Their chemical structure is characterized by an amino group bonded with an ester group, as shown in Table 2.

Typically, R_1_ and R_2_ are organic radicals or hydrogen. If R_2_ is hydrogen, it is possible to understand the target considering the functional group R_1_ [34]. It is possible to have:

- insecticides, when R_1_ is a methyl group;
- herbicides, when R_1_ is an aromatic group;
- fungicides, when R_1_ is a benzimidazole moiety.

Carbamates are also biocides for industry and household products for the control of household pests [35]. The common carbamate pesticides are aldicarb, carbofuran, fenoxycarb, carbaryl, ethienocarb, and fenobucarb [36].

As the organophosphates, carbamates are inhibitors of acetylcholinesterase activity, and therefore, their toxicity acts on the neurological system [37]. Exposure to carbamate pesticides increases the risk of non-Hodgkin’s lymphoma in humans since they inhibit and induce apoptosis of human natural killer (NK) cells and cytotoxic T lymphocytes that provide defense against tumors [38].

##### Pyrethrins and Pyrethroids

Pyrethrins are natural insecticides, in which active principle comes from the flowers of *Tanacetum cinerariaefolium,* also called *Chrysanthemum cinerariaefolium* or *Pyrethrum cinerariaefolium*. Their active constituents are esters of 2,2-dimethyl-3-(2-methyl-l-propenyl)-l-cyclopropanecarboxylic acid (chrysanthemic acid) and of 3-(2-methoxycarbonyl-l-propenyl)-2,2-dimethyl-l-cyclopropanecarboxylic acid (pyrethric acid). Six types were identified, as shown in Table 2.

Pyrethroids are synthetic compounds that are obtained by modifying the chrysanthemic acid moiety of pyrethrin I and esterifying the alcohols. They can be divided into [39]:

- First-generation pyrethroids: esters of chrysanthemic acid and one alcohol, having a furan ring and terminal side chain moieties.
- Second-generation pyrethroids: they have 3-phenoxybenzyl alcohols derivatives in the alcohol moiety and have some of the terminal side chain moieties replaced with a dichlorovinyl or dibromovinyl substitute and aromatic rings.

Pyrethroids are synthesized to increase the insecticidal power and decrease the sensitivity to air and light, compared to the pyrethrins.

Generally, in the air, the pyrethrins and many pyrethroids are rapidly degraded by sunlight, while they remain for a long time in the soil as they bind strongly to it [40]. Pyrethrins and pyrethroids may be found on leaves, fruits, and vegetables since they are sprayed directly onto crops and plants [41].

The pyrethrins and pyrethroids disrupt the sodium channels in the axons damaging the neurologic system [42]. They are toxic for insects but less harmful to humans. However, it was noted that the exposure of these pesticides can have respiratory effects such as cough or upper respiratory irritation after inhalation of dust or aerosol droplets; neurological effects such as headache or dizziness; gastrointestinal effects such as nausea and vomiting; and irritation and/or dermal effects [43]. Pyrethroids may cause cardiovascular problems [44].

#### 2.1.2. Biopesticides

**Figure 4:**
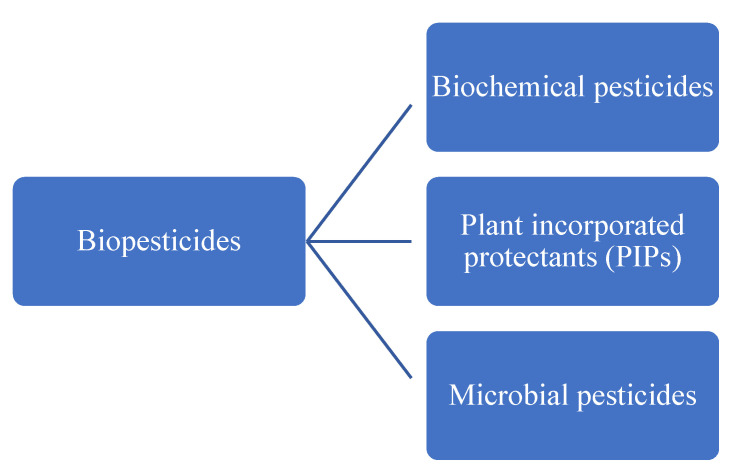
Classification of biopesticides.

Biopesticides are pesticides derived from nature (animals, plants, microorganisms, and minerals). They can be divided into three major classes (Figure 4) based on the type of active ingredient used, namely, biochemical, plant-incorporated protectants (PIPs), and microbial pesticides.

##### Biochemical Pesticides

They are natural compounds that control pests by nontoxic mechanisms. They can be extracted from natural sources or synthesized to have the same structure and function as the natural ones [45]. Semiochemicals are chemical compounds emitted by plants or animals. Pheromones, allomones, kairomones, and attractants are examples of these compounds. They are connected to the vital functions, such as feeding, mating, and egg-laying (ovipositing) of the pests [46]. Therefore, acting on their concentration can be exploited to influence the pest life cycle.

##### Plant-Incorporated Protectants

The plant-incorporated protectants (PIPs) can be produced by the plants themselves when the pest feeds on them. To force their production, the plants can be genetically modified introducing the gene acting on a specific pesticidal protein into the genetic material of the plant itself. In this way, the plant can synthesize the toxic compounds for selected pests [47].

##### Microbial Pesticides

Microbial pesticides include living organisms, such as bacteria, fungi, algae, and viruses, that control the pests. They suppress pests either producing toxic metabolites that cause damage and diseases or preventing the establishment of other microorganisms [48].

### 2.2. Classification by Targets

Pesticides can be classified by the roles that they play and the types of pests that they attack. The main classes are insecticides, herbicides, rodenticides, bactericides, and fungicides.

Based on their chemical structure, they may interact in a different way with pests and with different toxicity.

#### 2.2.1. Insecticides

Insecticides are chemical and biological compounds that attack and kill insects. Larvicides are specific insecticides that target the larval life stage of an insect.

These compounds are used in agriculture, horticulture, forestry, and gardening but are also used to control vectors, such as mosquitoes and ticks, which are involved in spreading human and animal diseases, such as dengue [49] and malaria [50].

The most-used insecticides belong to the classes of organophosphates, pyrethroids, and carbamates. They act on the nervous system of the victims, causing spasms, respiratory failure, and/or death. In Table 3, several insecticides used in agricultural soil are summarized.

#### 2.2.2. Herbicides

Herbicides are used to control and remove undesirable plants and weeds. These compounds are mainly applied in agricultural soils, before or during farming to maximize crop productivity. Herbicides are also used in forest management and in suburban and urban areas.

The modes of action depend on the chemical composition, and usually, they involve a plant enzyme or a biological system. In this way, the regular plant growth and developments are injured or disrupted, causing eventual plant death [51]. In Table 4, different herbicides used in agriculture soil are reported.

#### 2.2.3. Rodenticides

Rodenticides act to kill rodents, such as rats, mice, squirrels, and nutria; all these rodents can cause damage to crop, transmit disease, and cause ecological damage. Rodent infestations arise in a wide variety of situations: in agriculture soils, inside and around buildings, in sewers, in waste dumps, and/or in open areas.

Most rodenticides act as anticoagulants that interfere with blood clotting and cause death due to excessive bleeding. The rodenticide products are baits in block or paste form [52]. The most common rodenticides are summarized in Table 5.

#### 2.2.4. Fungicides

Fungicides are compounds that kill parasitic fungi or their spores. They permit the control of fungal infestations, especially occurring during the whole food supply. Indeed, fungicides are widely applied in the agricultural industry. Fungicides interfere with various biochemical processes within the fungal cytoplasm and mitochondria. They inhibit several enzymes and proteins involved for example in the lipid metabolism, fungal respiration, and production of adenosine triphosphate (ATP) [53].

Table 6 reports information on the most common fungicides.

## 3. Diffusion of Pesticides into the Environment and Their Toxicology

Pesticides aim to prevent, remove, and control harmful pests, but they may be harmful to the environment and human health. Their excessive use can give high concentrations of polluting substances in the environment. In the years, the World Health Organization ranked the pesticides and reported their toxicity and their effects on human health [54].

Through time, several pesticides have been banned in some countries due to their high toxicity. However, at the moment, their production and use go on, especially in developing countries.

### 3.1. Presence and Distribution into the Environment

The pesticides persist in the environment and may bioaccumulate and contaminate the food chain, affecting human health and the environment as a whole. Pesticide tends to conserve its molecular integrity and chemical, physical, and functional characteristics for a certain time after being released into the soil [55].

The parameter that can be considered to evaluate its persistence into the soil is the half-time (t_1/2_), that is, the time required for a compound to halve its initial concentration.

Pesticides with short half-times accumulate and persist less into the soil. By contrast, pesticides with long half-times are more persistent and this may increase the risk to contaminate the environment.

Altogether, they can be classified as [56]:

- nonpersistent pesticides, when t_1/2_ is lower than 30 days;
- moderately persistent, when t_1/2_ is in the range 30–100 days;
- persistent ones, whent_1/2_ is higher than 100 days.

Once present in the soil, pesticides may: (1) be adsorbed by soil particles; (2) be degraded by photochemical, chemical, and microbiological processes; and (3) move from soil to water.

#### 3.1.1. Adsorption by Soil Particles

Pesticide molecules can be adsorbed physically (Van der Waals forces) or chemically (electrostatic interactions) on the soil particles. The process can be described with the adsorption isotherms [57,58,59]. The adsorption constant is evaluated since it provides information about solute mobility. If pesticides have a low affinity for adsorption, they tend to spread more easily into the environment. Several soil parameters influence the adsorption process, namely soil organic matter content, clay content, clay mineralogy, and pH.

#### 3.1.2. Degradation Processes

The pesticides can be degraded and transformed into one or more metabolites through photochemical, chemical, and microbiological processes.

Photodegradation is an abiotic process induced by the absorption of light energy that leads to the decomposition of the polluting molecules. This process takes place with more difficulty in the soil, being a heterogeneous system, and it is influenced by soil properties. For example, photodegradation is more efficient with particles having a large size and a high specific area since they promote light diffusion [60]. Chemical and biological degradation occurs by reactions such as hydrolysis, oxidation, reduction, dehydrogenation, dehalogenation, decarboxylation, and condensation.

In the biodegradation process, pesticides are degraded by microbial organisms through metabolic or enzymatic action [61]. The evaluation of the kinetics of these reactions gives information on the persistence of pesticides.

#### 3.1.3. Leaching from Soil to Water

Leaching is the movement of pesticides within the soil. The soluble contaminants are carried by water downward through permeable soils. This phenomenon is responsible for the contamination of groundwater.

The extent of leaching is highly dependent on soil properties, pesticide physicochemical properties, formulation types, distribution of rainfall events or irrigation strategy, and hydrogeological processes [62].

### 3.2. Toxicity and Short- and Long-Term Damages

Several studies report the toxic effects on human health associated with the use of pesticides. Typically, the main routes of human exposure to pesticides are inhalation, ingestion, and dermal contact. Each compound has its toxicity, but the risk increases with increasing dosage and exposure time.

The WHO provides guidelines for the classification of pesticides, dividing them into five categories, and considering the lethal dose 50 (LD_50_) as a benchmark (Table 7) [54].

The LD_50_ value represents the dose required to kill half the tested population after a standardized test duration. The substance route can be given dermal and oral.

#### 3.2.1. Organochlorines

Considering the mode of action, the organochlorines damage the nervous systems, since they alter the ion channels. The main effects are hyperexcitability of brain, convulsions, tremor, hyperreflexia, and ataxia.

**Figure 5:**
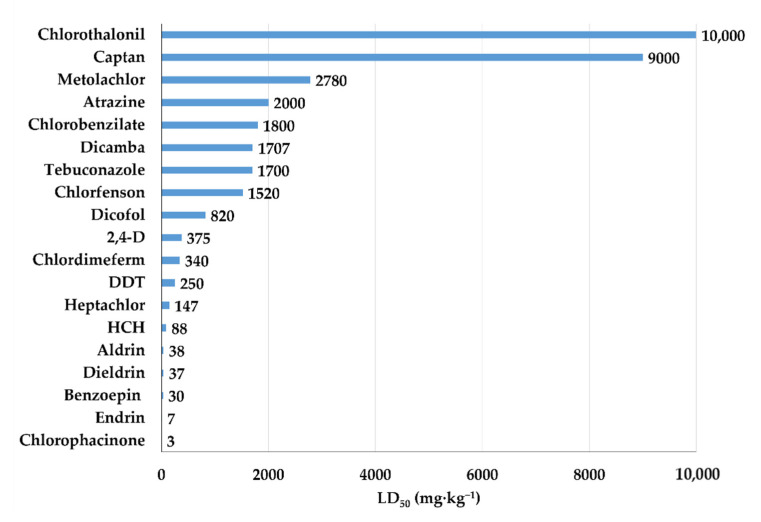
LD_50_ (mg·kg^−1^) of organochlorines for the rat.

Organochlorines have high toxicity and fall into the first classes of WHO classification (Figure 5).

It was noted that the major source for human exposure to organochlorines is food, particularly fish products [63]. The OCs are accumulated in fish muscle, and then, they become bioaccessible to humans during gastrointestinal digestion.

Significant concentrations of OCs were found on dust particles. This type of exposition causes cytotoxic effects on human skin, leading to the development of carcinoma [64].

#### 3.2.2. Organophosphates

**Figure 6:**
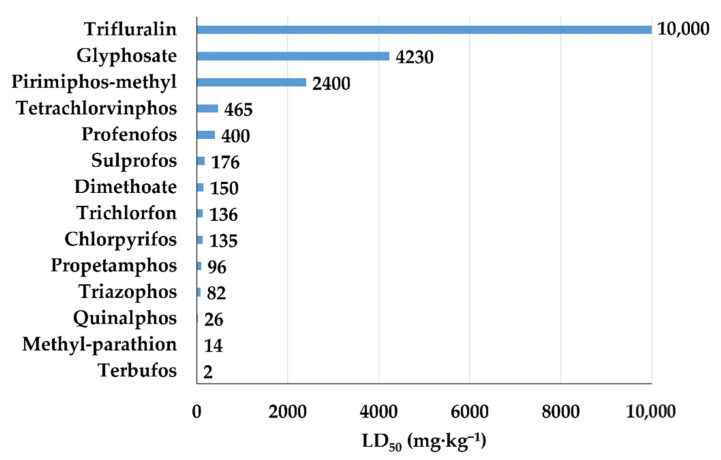
LD_50_ (mg·kg^−1^) of organophosphates for rat.

The organophosphates (OPs) are acetylcholinesterase inhibitors, which lead to having high levels of acetylcholine. The consequence is damage to several organs such as peripheral and central nervous systems, muscles, liver, pancreas, and brain. Most of them belong to the first classes (Ia, Ib, and II) of the WHO classification (Figure 6).

High doses of organophosphate cause acute intoxication, which leads to pancreatitis. This is due to the AChE-inhibition-induced cholinergic overstimulation, which leads to increased intraductal pressure and excess pancreatic enzyme secretion [65].

In the long term, one of the effects of organophosphate poisoning is a seizure disorder. Chuang et al. [66] have shown that the risk of seizure development is greater in patients with organophosphate poisoning than in healthy individuals.

#### 3.2.3. Carbamates

**Figure 7:**
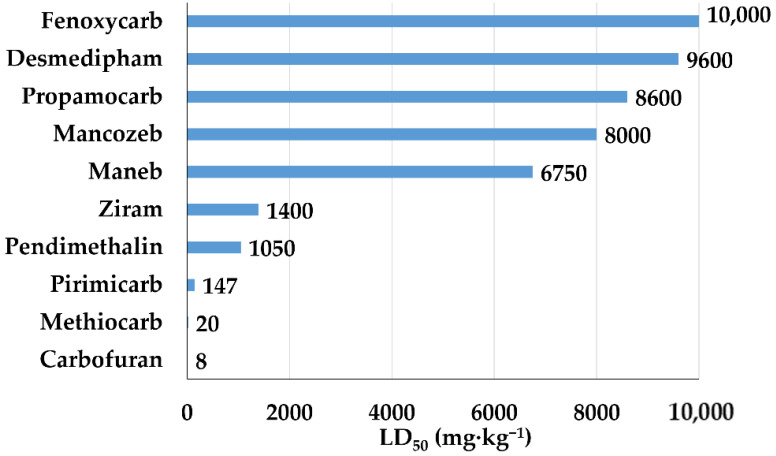
LD_50_ (mg·kg^−1^) of carbamates for rat.

The toxicity of several carbamates is such as to make them moderately and slightly hazardous, in some cases unhazardous, as can be seen from the LD_50_ values shown in Figure 7.

The carbamates act as acetylcholinesterase inhibitors and endocrine disruptors. Exposure to carbamates causes respiratory diseases.

Toxicological experiments have shown that prenatal and postnatal exposure to carbamates can affect fetal and child development, causing damage to the hippocampus [67,68].

It was noted that many fruits and beverages are contaminated by ethyl carbamate, a carcinogenic chemical causing the development of cancers in various animal species and rarely in humans. In particular, this pesticide can induce the production of reactive oxygen species, depurination of DNAs, and mitochondrial dysfunction [69,70].

#### 3.2.4. Pyrethroids and Pyrethrins

Pyrethrins and pyrethroids are less toxic to mammalian cells and less persistent in the environment than other pesticides. They can damage human health, mainly affecting the nervous system. The effects can be systemic, immunological, neurological, reproductive, developmental, genotoxic, and carcinogenic, and death [40]. The neurobehavioral functioning of fetuses and children is damaged by the exposition to these pesticides, since they have an immature nervous system, and their brain still has to grow and develop [71]. A correlation between pyrethroid exposure and the increased risk of diabetes in the adult population was found [72].

**Figure 8:**
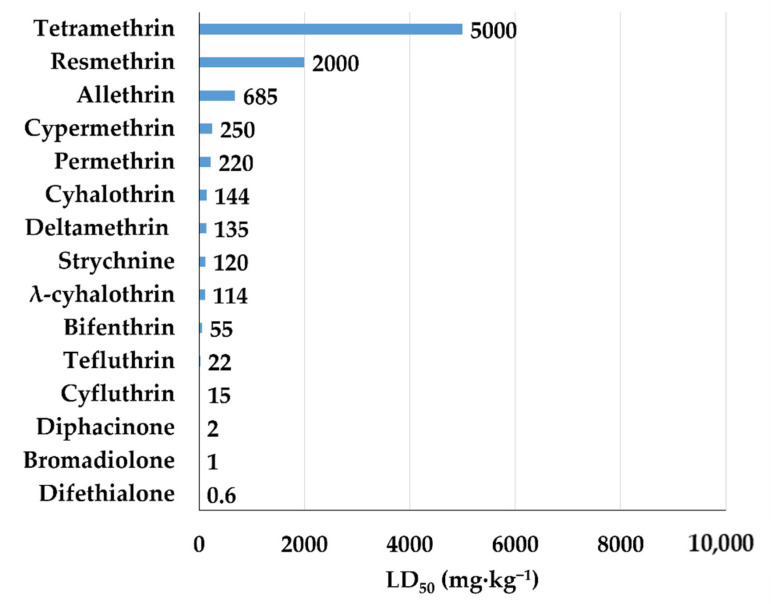
LD_50_ (mg·kg^−1^) of pyrethroids for rat.

In Figure 8, the LD_50_ value of several pyrenoids is shown. These compounds are more toxic than pyrethrins since they are synthesized with the purpose of increasing their insecticidal power.

## 4. Biological Techniques for Pesticide Removal

Bioremediation reduces pesticide contamination of agricultural soils by biodegradation processes via the metabolic activities of microorganisms. It is an efficient, cost-effective, and environment-friendly treatment.

During the bioremediation processes, the microorganisms use the pesticides as cosubstrates in their metabolic reactions together with other nutrients, thus eliminating them from the environment. The efficiency of these processes depends on the characteristics of pesticides, such as their distribution, their bioavailability, and their persistence in soil. It is necessary to promote the availability of pesticides to microorganisms: this is negatively affected by the adhesion of pesticides to soil particles and their low water solubility [73]. In addition, the soil characteristics and the environmental conditions, such as pH, water content, microbial diversity, and temperature, influence the bioremediation efficacy.

### 4.1. Mechanisms of Microbial Degradation

During biodegradation processes, pesticides are transformed into degradation products or completely mineralized by microorganisms, which use the pollutant compounds as nutrients for their metabolic reactions. A key role in the biotransformation mechanisms is carried out by enzymes, such as hydrolases, peroxidases, and oxygenases, that influence and catalyze the biochemical reactions.

The degradation process of pesticides can be divided into three phases, which can be summarized in:

- Phase 1: Pesticides are transformed into more water-soluble and less toxic products through oxidation, reduction, or hydrolysis reactions.
- Phase 2: The Phase-1 products are converted into sugars and amino acids, which have higher water solubility and lower toxicity.
- Phase 3: Conversion of the Phase-2 metabolites into less toxic secondary conjugates.

The microorganisms involved in the degradation process are bacteria or fungi, which may generate intra- or extra-cellular enzymes.

The degradation time is a relevant parameter to be assessed when a bioremediation activity is planned. It is typically interpreted by the first-order model [74], which depends on the pollutant concentration at the beginning and end of the process. This approach has limits because several parameters condition the process, such as microbial activity, temperature, water content, availability, and leaching of pesticide in the soil [61].

#### 4.1.1. Bacterial Degradation

In the years, several bacterial strains were identified as capable of degrading the pesticides present in the soils. Each bacterium has a specificity that makes it particularly suitable for a degradative process. The operative conditions, such as temperature, pH, water content, and types of pollutants, affect the adaptation, development, and role of a bacterial strain. Moreover, during the degradation process, metabolites can form and cause additional environmental problems, since they may be more difficult to remove than the original compound, and this must be considered a drawback. As an example, chlorpyrifos, an organophosphate used as an insecticide, is hydrolyzed by microorganisms, and the primary and major degradation product is 3,5,6-trichloro-2-pyridinol (TCP). TCP has greater water solubility than chlorpyrifos and causes widespread contamination in soils and aquatic environments. Few microorganisms can degrade the pesticide and its metabolite and among them the bacterium *Ochabactrum* sp. JAS2 is capable of hydrolyzing both compounds [75].

In many cases, the degradation is easier when a bacterial consortium is used compared to using an isolated pure culture [76,77]. In nature, the bacteria coexist and depend on each other for their viability. In the metabolic pathways of pesticide degradation, each bacterium can generate metabolites that may be used as a substrate by others.

#### 4.1.2. Fungal Degradation

The agricultural soils are populated by many fungi, which can be exploited to biodegrade pesticides. This class of microorganisms includes yeast, molds, and filamentous fungi. Fungal degradation is promoted by its capacity to produce many enzymes involved in degradative processes [78]. These microorganisms also can influence the soil properties, modifying soil permeability, and ion exchange capability.

Fungi can be better degraders than bacteria due to their characteristics, such as specific bioactivity, growth morphology, and high resistance even at high concentrations of pollutants.

A common approach is to use both fungi and bacteria to enhance degradation since fungi can transform pesticides into an easier and accessible form for bacteria [79].

#### 4.1.3. Enzymatic Degradation

Enzymatic biodegradation is due to the enzymes produced during the metabolic processes of microorganisms or plants. Enzymes are biological macromolecules that can catalyze biochemical reactions involved in pesticide degradation. These molecules act in the rate of reaction by lowering the activation energy of the reaction itself [80].

The main metabolic reactions, where they are involved, are oxidation, hydrolysis, reduction, and conjugation.

- Oxidation, which is the first step of the degradation of pesticides, consists of the transfer of an electron from reductants to oxidants. Oxygenase and laccase enzymes may be involved in this reaction. Oxygenases catalyze the oxidation reaction by incorporating one or two molecules of oxygen; laccases cleave the ring present in aromatic compounds and reduce oxygen to water and produce free radicals. During the reaction, heat or energy is generated, and it is utilized by microorganisms for their metabolic activities.
- Hydrolysis permits the cleavage of bonds of the substrate by adding hydrogen or hydroxyl groups from water molecules. The pesticide molecules are thus divided into smaller chain compounds than the original ones. Typical enzymes involved in the hydrolysis pathways are lipases, esterases, and cellulases. For example, Luo et al. [81] have identified and cloned an esterase gene from *Rhodopseudomonas palustris PSB-S* capable of decomposing several synthetic pyrethroids, such as fenpropathrin, and tolerates temperature and pH changes. The enzyme is involved in the key step of hydrolysis, namely the cleavage of the ester bond in the fenpropathrin compound.
- Reduction permits the transformation through reductive enzymes (nitroreductase).
- The conjugation reaction is carried out using existing enzymes, and it is typical of fungal biodegradation. It involves the addition of exogenous or endogenous natural compounds to facilitate the mineralization of pesticides. This process includes reactions such as xyloxylation, alkylation, acylation, and nitrosylation.

Table 8 reports some microorganisms able to degrade widely used pesticides.

#### 4.1.4. Mineralization

The mineralization process permits the degradation of pesticides into inorganic matter, namely, carbon dioxide, salts, minerals, and water. The microorganisms use the pesticide compounds as a source of nutrients.

Also in this case, the degradation is influenced by several factors, such as microbial species, soil characteristics, and type of pollutants. The mineralization rate depends on the concentration of microbial community; namely, a decrease in microbial population does not promote the degradation [88]. For example, chlorothalonil (CTN), an organochlorine fungicide, is degraded in CO_2_, but if the soil microbial community is reduced, several metabolites can form, which are more toxic, persistent, and mobile than CTN itself. This is due to the absence of actively-degrading groups or the decrease in soil biodiversity that leads to low microbial activity.

In glyphosate mineralization, the soil properties influence the mineralization process. Nguyen et al. [89] have tested agricultural soils, differing for some soil parameters, such as soil texture, soil organic matter content, pH, and exchangeable ions. By the univariate and multiple regression analysis, they have found the parameters that influence the glyphosate mineralization, namely: the cation exchange capacity, determined as the sum of exchangeable base cations and exchanges acidity (expressed as Al^3+^ and H^+^); the exchangeable base cations (expressed as Ca^2+^); and the available form of potassium, determined by ammonium lactate extraction. The low mineralization of glyphosate in soils with high exchangeable acidity could be due to either the formation of strong chemical bonds with the carboxylic or phosphonic acid groups of the glyphosate itself, reducing its bioavailability, or the toxic effects of exchangeable aluminum to soil microorganisms.

#### 4.1.5. Co-Metabolism

Co-metabolism is the biotransformation, through a series of reactions, of an organic compound that is not used to support microbial growth. The pesticides are transformed by microorganisms and enzymes into useful compounds for other biological, chemical, and physical transformations, and finally degraded thanks to this synergistic effect [90].

In the co-metabolic process, the involved enzymes can be:

- hydrolytic enzymes (esterases, amidases, and nitrilases);
- transferases (glutathione S-transferase and glucosyl transferases);
- oxidases (cytochrome P-450s and peroxidase);
- reductases (nitroreductases and reductive dehalogenases).

Ma et al. [91] have studied the co-metabolic transformation of imidacloprid (IMI), an insecticide, testing different types of substrates used as an energy source: carbohydrates and organic acids. *P. indica* CGMCC 6648 is the tested bacterium, capable of degrading IMI through the hydroxylation pathway, and forms two metabolites: one olefin and 5-hydroxy IMI.

### 4.2. Application of Microbial Remediation

The bioremediation techniques may be carried out in situ, ex situ, or on-site.

In the in situ approach, the treatment is carried out in the contaminated zone, and typically the process is aerobic. For this, it is necessary to provide oxygen to the soil. The main in situ techniques are:

- Natural attenuation, which exploits the microflora present in the polluted soil.
- Biostimulation, where the amounts and kind of nutrients to stimulate and promote the growth of indigenous microorganisms are optimized.
- Bioaugmentation, which is the addition of microbial strains or enzymes into the polluted soils.
- Bioventing, where oxygen is fed through unsaturated soil zones to stimulate the growth of indigenous microorganisms capable of degrading the contaminants.
- Biosparging, based on the injection of air under pressure into the saturated soil zone to increase the oxygen concentration and stimulate the microorganisms to degrade the pollutant.

These methods are very effective and cheap. Their main advantage is that the polluted soil is not moved.

Vice versa, in ex situ techniques, the contaminated soil is removed from polluted sites and transported to the site where the clean-up will occur. The main techniques are:

- Bioreactors, which treat the contaminated soil with wastewater to obtain a slurry and promote the microbial reactions capable of removing the pollutants.
- Composting, where the contaminated soil is mixed with amendments to promote the aerobic degradation of the pesticides. Landfarming and biopiles are included in this technique.

In on-site methods, the soil is removed and processed in the area close to the polluted site. For example, the landfarming treatment can also be effectuated on-site, reducing the operation cost comparing to the ex situ approach.

In all bioremediation processes, nutrients, oxygen, pH, water content, and temperature must be controlled to maximize removal efficiency.

#### 4.2.1. Natural Attenuation

Natural attenuation is a natural process where pollutants are degraded by indigenous microorganisms present in the soil. The natural processes include biological degradation, volatilization, dispersion, dilution, radioactive decay, and sorption of the contaminant onto the organic matter and clay minerals in the soil. For example, Guerin [92] demonstrated that endosulfan diol and endosulfan sulfate, two metabolites of insecticide endosulfan, are both mineralized through the microbial activity present in the contaminated soils.

#### 4.2.2. Biostimulation

The biostimulation process consists of the addition of nutrients (nitrogen, phosphorus, carbon, and oxygen) to promote the growth of the indigenous microorganisms. These nutrients are essential for the life of microorganisms and allow them to have energy, microbial population, and enzymes to degrade the pollutants.

Typically, nitrogen and phosphorus are added since they stimulate biodegradation and increase the diversity of microbial species. Betancur-Corredor et al. [93] have studied the degradation of DDT, DDD, and DDE, stimulating the microbial population and adding a surfactant. The number of nutrients supplied must be kept under control throughout the process, since a reduced or excessive quantity of stimulants could reduce microbial activity and their diversity.

Baćmaga et al. [94] have studied the degradation of tebuconazole in soil using the biostimulation process. The tebuconazole negatively influences the enzymatic activity and microbial proliferation; for this, its concentration in the soil must be reduced. A high concentration of pesticides leads to a decrease in the microbial population. The experimental tests by these authors have evaluated the effects of two different biostimulation substances (compost and bird droppings) on the remediation process. The results have shown that both substances had a positive effect on the development of soil microorganisms and enzymatic activity. The tebuconazole degradation was more intense in the soil fertilized with bird droppings than with compost.

#### 4.2.3. Bioaugmentation

The bioaugmentation process involves the inoculation of microbial consortia or single strains into the soil, by augmenting the microbial diversity. In this way, microorganisms with specific metabolic capabilities promote the biodegradation processes.

The concentration of pesticides in the soils is a parameter that conditions the process since high doses of pesticides inhibit the vital functions of soil microorganisms. Doolotkeldieva et al. studied the bacterial degradation of pesticide-contaminated soils in dumping zones. In a preliminary study [10], Doolotkeldieva et al. found that several bacterial strains were present in the studied soils. Then, they tested the degradation of aldrin, that is a diffused chlorinated hydrocarbon pesticide. The results have demonstrated that bacteria strains with specific genes (cytochrome P450), namely *Pseudomonas fluorescens* and *Bacillus polymyxa*, were capable of degrading aldrin in a relatively short time. The selection of specific bacterium, the optimization of soil conditions such as temperature, pH, and the nutrients available in the soil, were used for the development of the next experimental tests. In particular, mesocosms were set up with soil contaminated with several pesticides and inoculated with the microbial consortium [76].

In contaminated soil, the pesticide concentrations can vary at different depths since the pesticides leach into the subsurface of soil and adsorb on the soil particles, making them less bioavailable. Odukkathil and Vasudevan [95] have evaluated the bioaugmentation treatment in an experimental test set up in a glass column with a volume of 4500 cm^3^. The results have shown that the pesticide concentrations in the bottom soil were high, due to the downward drift of pesticides during the water seepage, whereas the low concentrations in the central soil could be due to higher microbial activity favoring the degradation.

##### Application of Natural Attenuation, Biostimulation and Bioaugmentation

Several studies have been conducted to evaluate and compare the biodegradation of pesticides through natural attenuation, biostimulation, and bioaugmentation strategies. For example, Bhardawaj et al. [96] have analyzed the biodegradation of atrazine with three different techniques. Each mesocosm was set up with 100 kg of soil and contaminated with a concentration of atrazine equal to 300 mg·kg^−1^ of soil. They have found that despite the natural attenuation indicating that the soil microbiome possessed an inherent potential for atrazine biodegradation, the natural process was slow. Conversely, with biostimulation and bioaugmentation treatments, the atrazine was completely removed after 35 days. Moreover, the bioaugmentation strategy was more rapid than biostimulation since after 21 days the pollutant was degraded. Authors recommend this method for the treatment to be fast and cheap.

The bioremediation of contaminated soils might be more efficient when coupling bioaugmentation and biostimulation treatments [97]. Raimondo et al. [98] have tested 1 kg mesocosms polluted with lindane at a concentration equal to 2 mg·kg^−1^ of soil. They have demonstrated that the removal of lindane increases and the half-life of pesticide can be reduced using simultaneously bioaugmentation and biostimulation.

#### 4.2.4. Bioventing

Bioventing is an in situ bioremediation technique that promotes the degradation of organic pollutants adsorbed to the soil. The microbial activity is enhanced by the introduction of air or oxygen flow, and nutrients into the unsaturated zone of soil through specifically constructed wells into contaminated soils. The ventilation is light, and it is necessary to provide the only oxygen needed to sustain microbial activity and avoid the volatilization of contaminants.

**Figure 9:**
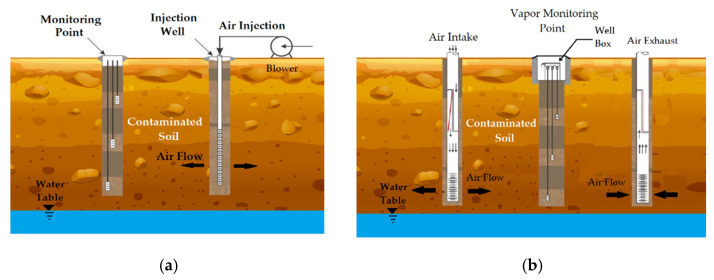
Scheme of bioventing process: (**a**) active technology and (**b**) passive technology.

Bioventing can be realized in active or passive mode, with regards to the aeration: in the first case, the air is driven into the soil with a blower, while, in the passive method, the gas exchange through the vent wells occurs only by the effect of atmospheric pressure. The schemes of the two aeration methods are shown in Figure 9.

Bioventing remediation may last from 6 months to 5 years, depending on the kind and concentration of contaminant, biodegradation rates, and characteristics of soil, such as permeability and water content.

#### 4.2.5. Biosparging

In the biosparging technique, the biodegradation process occurs by stimulating the indigenous microorganisms through the injection of air in groundwater to increase the oxygen concentration. The method is similar to bioventing, except that in the biosparging the air is injected in the saturated zone. This can cause upward movement of volatile organic compounds to the unsaturated zone to promote biodegradation. Two parameters that influence the effectiveness of the process are (1) soil permeability, which determines pollutant bioavailability to microorganisms, and (2) pollutant biodegradability.

#### 4.2.6. Composting

Composting is an approach for the bioremediation of pesticides. It consists of mixing the contaminated soil with nonhazardous organic amendments to promote the development of bacterial and/or fungi population, able to degrade the pesticides through a co-metabolic pathway.

This approach is particularly indicated when pesticide concentration is low. In composting, the microbial bioaccessibility to the pollutant is crucial. For this reason, it is important to control the water content, soil composition, and properties of the added amendment.

In contaminated soils, biochar can be used as an amendment to promote the degradation processes. Biochar is black carbon produced by the thermal conversions of biomass under limited oxygen conditions (gasification) or in the absence of oxygen (pyrolysis). It is characterized by high porosity and a large surface area; these two properties promote the adsorption of pesticides. Moreover, biochar is a carbon source that stimulates microbial activity, promoting biodegradation. It has been noted that biochar application increases the soil water holding capacity and improves aeration conditions in soil [99]. Sun et al. [100] have studied its application for the biodegradation of tebuconazole. This allowed the immobilization of *Alcaligenes faecalis WZ-2*, the bacterial strain involved in the degradation process.

Composting can be carried out with two techniques: landfarming and biopiles.

##### Landfarming

Landfarming is an aerobic bioremediation process carried out for a long time. Contaminated soils are transported to a landfarming zone, incorporated into the soil surface over large areas, and periodically tilled to aerate the mixture. The kinetics of the degradation process is slow and may also take years. During the process, leaching and/or volatilization of toxic compounds (original compound and metabolites) must be controlled or prevented. To avoid any risk of infiltration, a waterproof cover must be in place on the soil before the start of the treatment. This treatment is applied especially to soils contaminated with a mixture of pollutants.

##### Biopiles

Biopiles are piles of contaminated soil, equipped with a piping system that permits the aeration. During the process, air or oxygen is sent, and a solution containing nutrients is applied to the soil surface to stimulate microbial activity. The parameters that influence the process are water contents, temperature, pH, and concentration of nutrients and oxygen that must be controlled to enhance biodegradation.

#### 4.2.7. Slurry Bioreactors

In slurry bioreactors, the contaminated soil is mixed with wastewater up to obtain a slurry with aqueous suspensions between 10% and 30% w·v^−1^. The bioreactor can be operating under aerobic or anaerobic conditions. Baczynski et al. [101] studied the anaerobic biodegradation of organochlorine pesticides. They used methanogenic granular sludge as inoculum for anaerobic treatment of soil contaminated by γ-hexachlorocyclohexane, methoxychlor, o,p’- and p,p’-DDT. The pollutants were removed to a good extent at all tested temperatures (12, 22, and 30 °C) without a lag phase. Low temperature reduces the removal rates of pesticides. This might be due to the reduction of desorption rates of slowly desorbing fractions of these pollutants. In another study [102], the same authors found that the rapidly desorbing fractions were not a good indicator for the evaluation of the bioremediation process. Instead, the determination of slowly desorbing fractions can be better used for this purpose.

### 4.3. In-Field Applications

At present, few studies report information on real case studies.

Table 9 summarizes some examples. Unfortunately, the findings and results of large-scale remediation are usually neither published nor widely publicized, limiting the knowledge of experiences on real cases. A similar situation occurs for the costs of the clean-up.

## 5. Legislation

The necessity of having sustainable food production and a reduction, or even a ban, of the use of pesticides, has made it so that each country in the world is committed to implement measures and laws in this regard. Dealing with the topic at the world level, the Food and Agriculture Organization of the United Nations (FAO) and the World Health Organization (WHO) have released a pair of updated guidelines on pesticide legislation and labeling. A code for pesticide legislation has been published in 2020 [107], aiming to guide governments that seek to review, update, or design national pesticide legislation.

### 5.1. European Union

In the European Union, the sustainable use of pesticides is ruled by Directive 2009/128/EC [108]. The aim is to reduce the risks and impacts of these chemicals on health and the environment.

Moreover, Regulation (EC) 1107/2009 [5] contains the criteria and rules to authorize the active substances of pesticides and plant protection products (PPPs) and their placement on the European market. Pesticides cannot be marketed and used if they have not been previously approved and authorized by this procedure.

The institution which deals to evaluate active substances contained in pesticides is the European Food Safety Authority (EFSA). EFSA supports the European Commission, the European Parliament, and the EU member states in taking effective and timely risk management decisions to ensure the protection of the health and the safety of the food and feed chain. Each EU member state evaluates and authorizes the products with national laws.

In the member states, Regulation (EC) No. 1107/2009 [5] has been underpinned by national laws through the formulation of more precise stipulations at the national level. In particular, the National Action Plans include quantitative objectives, targets, measures, and timetables, and they are to be reviewed at least every 5 years. The first National Action Plans were communicated to the Commission in 2012, and a report by the EU Commission [109] has shown that in 2017 all Member States had plans in place.

### 5.2. United States of America

In the United States, the regulation and monitoring of pesticides are ruled by three government agencies:

- The U.S. Environmental Protection Agency (EPA) registers and approves the use of pesticides and establishes the maximum amounts of residues that are permitted inside or on food.
- The U.S. Department of Agriculture (USDA) is responsible for the enforcement of pesticide tolerances primarily in meat, poultry, and certain egg products.
- The U.S. Food and Drug Administration (FDA) is responsible for the enforcement of pesticide tolerances in other food categories, both domestic and imported ones.

The EPA regulates the use of pesticides considering two federal statutes: (1) the Federal Insecticide, Fungicide, and Rodenticide Act (FIFRA), enacted in 1947 and updated with new amendments [110]. It regulates the registration, distribution, and use of pesticides, including the toxicology, environmental fate, and ecotoxicology and residue chemistry; (2) the Federal Food, Drug, and Cosmetic Act (FD&C). It establishes tolerances or safe levels of pesticide residues in raw agricultural commodities. The updated act is FD&C 2021 [111].

The EPA works cooperatively with the state agencies to review pesticide safety data, register products, educate professional applicators, monitor compliance, and investigate pesticide problems. State governments develop regulations that are stricter than the federal regulation given by EPA.

### 5.3. India

In India, the pesticide regulation was applied for the first time in the insecticide act [112], by the Ministry of Agriculture, Department of Agriculture and Cooperation in 1968. Since 1971, the insecticide rules [113] have been in force: they regulate the import, sale, transportation, manufacture, and use of persistent pesticides.

Under this act, all insecticides must be recorded by the Central Insecticides Board (CIB) and Registration Committee (RC). The CIB and RC scrutinize and periodically review all pesticides, before authorizing their sale and use. Moreover, they have the authority to ban environmentally threatened pesticides even after their registration.

### 5.4. Regulation in Other Countries

The regulations on pesticides in other countries are more recent.

In China, the Ministry of Agriculture is responsible for the enforcement of the Law on Quality and Safety of Agricultural Products and the Regulation on the Control of Agrochemicals. The National Food Safety Standard—Maximum Residue Limits for Pesticides in Foods [114] was approved in 2012 and updated several times in the following years to add other maximum residue limits of pesticides in foods, until the current version released in February 2021 [115].

In Japan, the pesticide regulation is contained in the Food Safety Basic Law, enacted in 2003 [116]. This law sets the principles for developing a food safety regime and defines the maximum acceptable concentration limits of residual pesticides. A limit of 0.01 mg·kg^−1^ of soil applies to compounds for which tolerance limits have not been established.

## 6. Conclusions

After the Second World War, the use of pesticides and plant protection products has grown heavily, both in developed and developing countries. Unfortunately, all these compounds are toxic to different extents and impact human health and the environment. Moreover, many of them are persistent; that is to say, their degradability is very limited and occurs for long times.

Soil bioremediation for their removal can be carried out exploiting either specific or indigenous microorganisms (bacteria and fungi), or enzymatic degradation.

While at a laboratory scale, many findings on soil bioremediation are available in the literature, few data on real-scale activities can be found. Unfortunately, this is mainly due to the poor cooperation among research laboratories, local authorities imposing a given soil clean-up, and companies involved in the sector of bioremediation in soils polluted with pesticides.

It would be beneficial that more and more this cooperation becomes united, to disseminate the experiences and results. Moreover, the cost data are lacking, too.

As for other pollutants, when required, the pesticide removal must take into account the chemical and toxicological characteristics of the compounds, without disregarding the national legislation. To this purpose, it must be outlined that several countries are still lacking in legislative acts, and this is the main drawback when polluted areas must be remediated.

## Figures and Tables

**Table 1:** Reviews on pesticides.

Topic	References
Pesticide diffusion in the environment	[13,14]
Toxic effects on living organisms	[13,15,16]
Legislation	[14]
Physical techniques for pesticide degradation	[17,18]
Chemical techniques for pesticide degradation	[16,17,18]
Biological techniques for pesticide degradation	[17,18,19,20,21,22]
Microorganisms capable of degrading pesticides	[13,19,21,22,23]
Enzymatic degradation	[24]
Economic analysis	[17]
Degradation of organochlorine pesticides	[14,16]
Degradation of herbicides.	[13]
Monitoring of pesticide clean-up	[20]

**Table 2:** Chemical composition of the pesticides.

Group	Chemical Structure
Organochlorines	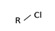
Organophasphates	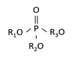
Carbamates	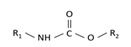
Pyrethrins and pyrethroids	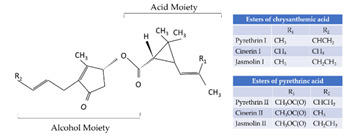

**Table 3:** Classification of the most common insecticides used in agricultural soil.

Name	Pesticide Class	Chemical Structure	Mode of Action
DDT	Organochlorine	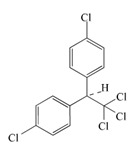	Interaction with sodium ion channels in neurons, causing their inactivation, which leads to spasms and eventual death.
Cyfluthrin	Pyrethroid	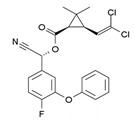	Interference with nerve signaling by inhibition of the membrane sodium channel systems.
Tefluthrin	Pyrethroid	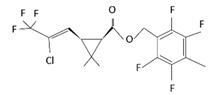	Interruption of functioning of the nervous system, interfering with sodium channels.
Aldicarb	Carbamate	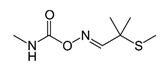	Inhibition of cholinesterase prevents the breakdown of acetylcholine in the synapse. It leads to respiratory failure.
λ-cyhalothrin	Pyrethroid	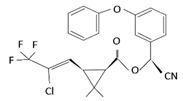	Interference with nerve signaling by inhibition of the membrane sodium channel systems.
Permethrin	Pyrethroid	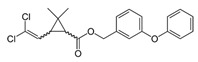	Interference with sodium channels disrupts the function of neurons and causes muscles to spasm, culminating in paralysis and death.
Terbufos	Organophosphate	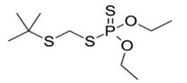	Inactivation of acetylcholinesterase by phosphorylation of the hydroxyl group of serine present at the active site of the enzyme.
Chlorpyrifos	Organophosphate	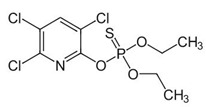	Disruption of nervous systems by inactivation of acetylcholinesterase.
Methyl-parathion	Organophosphate	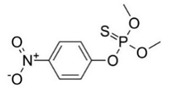	Disruption of nervous systems by inactivation of acetylcholinesterase.
Dimethoate	Organophosphate	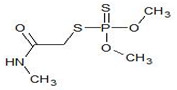	Disruption of nervous systems by inactivation of acetylcholinesterase.
Carbofuran	Carbamate	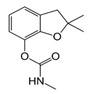	Disruption of nervous systems by inactivation of acetylcholinesterase.

**Table 4:** Classification of the most common herbicides used in agricultural soil.

Name	Pesticide Class	Chemical Structure	Mode of Action
Glyphosate	Organophosphate	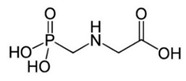	Disruption of shikimic acid pathway through inhibition of the enzyme 5-enolpyruvylshikimate-3-phosphate synthase.
Atrazine	Organochlorine	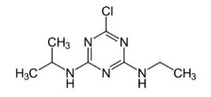	Inhibition of the photosynthetic pathway, specifically the photosystem II.
2,4-D	Organochlorine	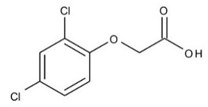	Imitation of plant growth hormone auxin and uncontrolled cell division in vascular tissue, leading to uncontrolled growth and eventually death of plants.
Dicamba	Organochlorine	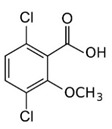	Imitation of plant growth hormone auxin and uncontrolled cell division in vascular tissue, leading to uncontrolled growth and eventually death of plants.
Trifluralin	Organophosphate	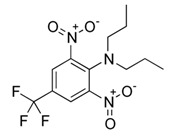	Inhibition of cell mitosis, acting on the meristems and tissues of underground organs.
Metolachlor	Organochlorine	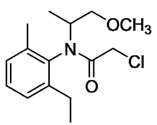	Inhibition of elongases and the geranylgeranyl pyrophosphate cyclases, important in the synthesis of long-chain fatty acids.
Cyanazine	Organochlorine	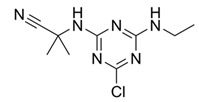	Inhibition of the photosynthetic pathway, specifically the photosystem II.

**Table 5:** Classification of the most common rodenticides.

Name	Pesticide Class	Chemical Structure	Mode of Action
Chlorophacinone	Organochlorine	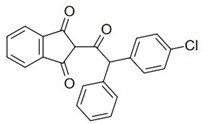	Anticoagulant agent depressing hepatic synthesis of prothrombin and clotting factors VII, IX, and X, inducing internal hemorrhage.
Diphacinone	Pyrethroid	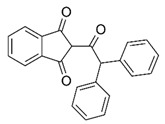	Inhibition of vitamin K epoxide reductase complex 1, which is an essential enzyme for activating.
Bromadiolone	Pyrethroid	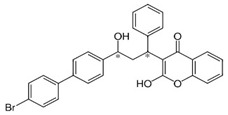	Anticoagulant agent depressing hepatic synthesis of prothrombin and clotting factors VII, IX, and X, inducing internal hemorrhage.
Difethialone	Pyrethroid	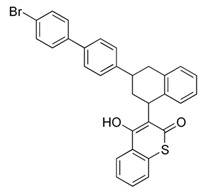	Anticoagulant agent depressing hepatic synthesis of prothrombin and clotting factors VII, IX, and X, inducing internal hemorrhage.
Strychnine	Pyrethroid	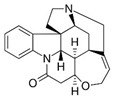	Inhibition of glycine neurotransmitters, as glycine and acetylcholine.

**Table 6:** Classification of the most common fungicides used in agricultural soil.

Name	Pesticide Class	Chemical Structure	Mode of Action
Mancozeb	Carbamate	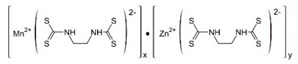	Inhibition of enzyme activity in fungi by forming a complex with metal-containing enzymes.
Chlorothalonil	Organochloride	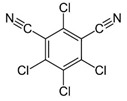	Reductor and deactivator of glutathione.
Captan	Organochloride	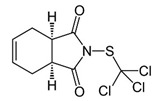	Reduction of enzymatic activity.
Maneb	Carbamate	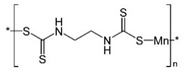	Reaction and inactivation of sulfhydryl groups of amino acids and enzymes, causing damage to lipid metabolism, respiration, and production of adenosine triphosphate.
Ziram	Carbamate	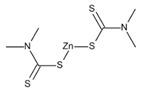	Creation of chemical barrier between the plant and a fungus.
Tebuconazole	Organochloride	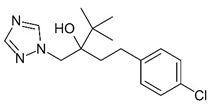	Inhibitor of sterol 14α-demethylase

**Table 7:** Criteria for classification of pesticides.

Class	Characteristics	LD_50_ for the Rat (mg·kg^−1^ Body Weight)	
		Oral	Dermal
Ia	Extremely hazardous	<5	<50
Ib	Highly hazardous	5–50	50–200
II	Moderately hazardous	50–2000	200–2000
III	Slightly hazardous	>2000	>2000
U	Unlikely to present acute hazard	>5000	>5000

**Table 8:** Microorganisms capable to degrade several pesticides.

Target	Pesticide	Microorganism	References
Insects	Chlorpyrifos	*Ochrobactrum* sp. JAS2	[75]
	Cypermethrin	*Bacillus subtilis*	[82]
	DDT	*Fomitopsis pinicola* and *Ralstonia pickettii*	[79]
	Deltamethrin	*Streptomyces rimosus*	[74]
	Fentopropathrin	*Rhodopseudomonas palustris PSB-S*	[81]
	Phorate	*Brevibacterium frigoritolerans, Bacillus aerophilus* and *Pseudomonas fulva*	[77]
Herbs	Acetochlor	*Tolypocladium geodes* and *Cordyceps*	[11]
	Glyphosate	*Fusarium*	[83]
	Glyphosate and its metabolites	*Pseudomonas fluorescens*	[84]
	Penoxsulam	*Aspargillus flavus* and *Aspargillus niger*	[85]
Fungi	Epoxiconazole and fludioxonil	*Pseudomonas, Rhodobacter, Ochrobactrum, Comamonas, Hydrogenophaga, Azospirillum, Methylobacillus,* and *Acinetobacter*	[86]
	Tebuconazole	*Serratia marcescens*	[87]

**Table 9:** Some examples of case studies.

Bioremediation Technique	Pesticides	Description	References
Landfarming	Hexachlorocyclohexane (HCH) isomers (insecticides)	Contaminated soil with HCH isomers (>5000 mg·kg^−1^) derived from lindane production was studied in the field for 11 months, setting up two plots (each 2 m × 10 m). The α- and γ-HCH isomers were decreased by 89 and 82% of the initial concentration, respectively. The concentration of the most persistent β-isomer remained essentially unaffected.	[103]
Bioaugmentation	Myclobutanil, tetraconazole, and flusilazole.	Experimental tests were conducted on vineyard plots. In the crops, an agricultural formulation of pesticides by foliar spray was applied. After one h of pesticide application, vines were sprayed with a suspension of four *Bacillus* strains. DR-39, CS-126, TL-171, and TS-204 were tested. Residue analysis of field samples showed 87.4 and >99% degradation of myclobutanil and tetraconazole, respectively, by the strain DR-39, and 90.8% degradation of flusilazole by the strain CS-126 after 15−20 days of treatment.	[104]
Bioaugmentation	DDT	The bioremediation process was studied in 12 experimental plots, including greenhouse and open field soils. Each plot (area of 6 m^2^) was inoculated with *Stenotrophomonas* sp. DDT-1 supplemented with 2% yeast powder. The results have shown that this microorganism is efficient for DDT degradation and does not adversely affect soil microbial activity.	[105]
Biostimulation	Organochlorine pesticides: toxaphene; DDT; DDE; DDD; endosulfan II; γ-chlordane; α-chlordane; dieldrin.	The Borello Property is a 14 acre area treated with soil amendment to help the indigenous bacteria to metabolize the pesticides. For the analysis, the area was divided into zones and in each of them, the soil samples were collected from four soil depths (0.5, 1, 1.5, and 2 ft). At the end of the test, OCPs were not detected; toxaphene, DDT, and DDE were detected in a single sample; dieldrin was detected in five samples at concentrations ranging from 1.2 to 1.8 μg·kg^−1^.	[106]
Biostimulation	Organochlorine pesticides: toxaphene; DDT; DDE; DDD; endosulfan II; γ-chlordane; α-chlordane; dieldrin.	The Mantegani Property is a 0.8 acre area treated with soil amendment to help the indigenous bacteria to metabolize the pesticides. High concentrations of DDT and dieldrin were present. After treatment, DDT was degraded by 97% and dieldrin by 73%, while the concentrations of other OCPs were below their preliminary remediation goals.	[106]

## Data Availability

Not applicable.
